# Z-score mapping for standardized analysis and reporting of cardiovascular magnetic resonance modified Look-Locker inversion recovery (MOLLI) T1 data: Normal behavior and validation in patients with amyloidosis

**DOI:** 10.1186/s12968-019-0595-7

**Published:** 2020-01-20

**Authors:** Riccardo Kranzusch, Fabian aus dem Siepen, Stephanie Wiesemann, Leonora Zange, Sarah Jeuthe, Tiago Ferreira da Silva, Titus Kuehne, Burkert Pieske, Christoph Tillmanns, Matthias G. Friedrich, Jeanette Schulz-Menger, Daniel R. Messroghli

**Affiliations:** 1 Department of Internal Medicine – Cardiology, Deutsches Herzzentrum Berlin, Augustenburger Platz 1, 13353 Berlin, Germany; 2 Department of Internal Medicine and Cardiology, Campus Virchow-Klinikum, Charité – Universitätsmedizin Berlin, Berlin, Germany; 3 Department of Cardiology, Angiology and Pneumology, Universitätsklinikum Heidelberg, Heidelberg, Germany; 4 Experimental and Clinical Research Centera joint cooperation between the Charité Medical Faculty and the Max-Delbrueck Center for Molecular Medicine and HELIOS Hospital Berlin Buch, Berlin, Germany; 5 German Center for Cardiovascular Research (DZHK), partner site Berlin, Berlin, Germany; 6 Max-Delbrück-Center for Molecular Medicine, Berlin, Germany; 7 Department of Congenital Heart Disease and Paediatric Cardiology, Charité – Universitätsmedizin Berlin, Berlin, Germany; 8 Institute for Imaging Science and Computational Modelling, Charité – Universitätsmedizin Berlin, Berlin, Germany; 9 Diagnostikum Berlin, Berlin, Germany; 10 Departments of Medicine and Diagnostic Radiology, McGill University, Montréal, Canada; 11 Department of Cardiology and Nephrology, HELIOS-Klinikum Berlin Buch, Berlin, Germany

**Keywords:** Myocardial disease, Tissue analysis, Magnetic resonance imaging, T1 mapping, Standardization, Z-score, Amyloidosis

## Abstract

**Background:**

T1 mapping using modified Look-Locker inversion recovery (MOLLI) provides quantitative information on myocardial tissue composition. T1 results differ between sites due to variations in hardware and software equipment, limiting the comparability of results. The aim was to test if Z-scores can be used to compare the results of MOLLI T1 mapping from different cardiovascular magnetic resonance (CMR) platforms.

**Methods:**

First, healthy subjects (*n* = 15) underwent 11 combinations of native short-axis T1 mapping (four CMR systems from two manufacturers at 1.5 T and 3 T, three MOLLI schemes). Mean and standard deviation (SD) of septal myocardial T1 were derived for each combination. T1 maps were transformed into Z-score maps based on mean and SD values using a prototype post-processing module. Second, Z-score mapping was applied to a validation sample of patients with cardiac amyloidosis at 1.5 T (*n* = 25) or 3 T (*n* = 13).

**Results:**

In conventional T1 analysis, results were confounded by variations in field strength, MOLLI scheme, and manufacturer-specific system characteristics. Z-score-based analysis yielded consistent results without significant differences between any two of the combinations in part 1 of the study. In the validation sample, Z-score mapping differentiated between patients with cardiac amyloidosis and healthy subjects with the same diagnostic accuracy as standard T1 analysis regardless of field strength.

**Conclusions:**

T1 analysis based on Z-score mapping provides consistent results without significant differences due to field strengths, CMR systems, or MOLLI variants, and detects cardiac amyloidosis with the same diagnostic accuracy as conventional T1 analysis. Z-score mapping provides a means to compare native T1 results acquired with MOLLI across different CMR platforms.

## Background

The ability to extract a multitude of information from soft tissues in a non-invasive manner has allowed cardiovascular magnetic resonance (CMR) to become the preferred imaging modality for tissue characterization in many organs. With late gadolinium enhancement (LGE) [1] and T2-weighted short tau inversion recovery (STIR) [2] imaging, dedicated variants of conventional CMR techniques were introduced to cardiac applications to detect *regional* myocardial lesions and edema, respectively, establishing CMR as an essential diagnostic tool in myocardial diseases. By design these techniques are optimized to generate maximum contrast between normal and abnormal areas of the myocardium to facilitate qualitative (visual) assessment. The introduction of single-breathhold pulse sequences for cardiac T1 mapping such as MOLLI [3] for clinical CMR systems has added an additional layer of information, as they enable a direct quantitative assessment of both focal or global signal intensities in clinical routine. T1 mapping allows for evaluating myocardial tissue properties by deriving absolute values of the magnetic tissue property T1 from a specific region or the entire myocardium, which then can be compared to local reference values derived from healthy controls. Thus, T1 mapping intrinsically carries the potential to also detect *diffuse* myocardial disorders. A multitude of studies have proven the validity of this concept for various myocardial diseases and conditions including cardiac amyloidosis [4], Fabry’s disease [5], myocarditis [6], and diffuse myocardial fibrosis [7]. The Heart Failure Association of the European Society of Cardiology recently identified parametric mapping as one of six areas of innovative imaging methods with the potential to revolutionize the assessment of heart failure [8].

Various pulse sequence schemes have been developed for clinical T1 mapping [9–11]. Depending on their technical approach, their accuracy and precision vary, resulting in significantly different reference ranges for myocardial T1 [12]. Moreover, results are confounded by external factors such as field strength and manufacturer-specific hardware design of the CMR system. Therefore it has been recommended that each site should generate their own local reference ranges from site-specific T1 measurements of healthy controls or of patients without other signs or history of myocardial disease [13, 14]. However, this approach does not solve the problem of results not being directly comparable from different sites or CMR systems. Moreover, the lack of a uniform reference range is perceived as a barrier for the translation of findings from studies that were performed with other acquisition schemes, and thus seriously limits further development and clinical dissemination of T1 mapping.

In biostatistics, Z-scores are multiples of standard deviations (SD) from the mean of a normally distributed population [15]. In clinical medicine they are typically used to compare a quantitative test result to non-intuitive reference data, e.g. for gender-, age- and size-specific dimensions of the aortic root in children [16]. The aim of our study was to apply Z-scoring to T1 mapping in order to standardize reporting of results. We hypothesized that the use of Z-scores would result in universal T1 results that are comparable and clinically meaningful irrespective of the mapping variant, CMR system, and field strength used.

## Methods

The study consisted of two parts. First (evaluation step), Z-score mapping was applied to T1 maps obtained from healthy subjects (as confirmed by normal findings on electrocardiogram (ECG), transthoracic echocardiography, and cardiopulmonary exercise test; *n* = 15) in order to evaluate the variability of results in normal controls. All participants underwent T1 mapping with 2 to 3 different MOLLI schemes on four CMR systems from two different manufacturers at three sites (11 T1 maps for each subject). Second, a validation step was performed. Z-score mapping was applied to T1 maps from patients with cardiac transthyrein (ATTR) amyloidosis (as confirmed by endomyocardial biopsy and/or bone marrow scintigraphy) who underwent T1 mapping with one MOLLI scheme at 1.5 T (*n* = 25) or 3 T (*n* = 13) at a fourth site using normal data from healthy subjects who were scanned on the same 1.5 T system (*n* = 14) or 3 T system (*n* = 16) at the same site with the same MOLLI scheme.

### Image acquisition – evaluation step

Fifteen healthy subjects (25 ± 4 years; 7 male) underwent multiple T1 mapping CMR studies in mid-cavity short axis orientation at three sites within 1 week. In order to achieve reproducible positioning of the imaging planes, the “systolic 3-of-5” approach was used [17]. At site 1, T1 mapping was performed on a 1.5 T CMR system (Achieva, software release 5.1.8; Philips Healthcare, Best, The Netherlands) and on a 3 T (Ingenia, software release 5.1.8, Philips Healthcare) system using MOLLI 3b (3b) 3b (3b) 5b, MOLLI 5b (3b) 3b, and MOLLI 5 s (3 s) 3 s. At site 2, T1 maps were acquired on a 1.5 T Siemens Avanto (software release D13B; Siemens Healthineers, Erlangen, Germany) system using the same 3 MOLLI variants. At site 3, MOLLI 3b (3b) 3b (3b) 5b and MOLLI 5b (3b) 3b were obtained similar to sites 1 and 2 from a 3 T (Skyra, software release E11, Siemens Healthineers); MOLLI 5 s (3 s) 3 s was not available on this system. Only product mapping packages were used for MOLLI T1 mapping. Scanning was performed at each site by one local operator with >5 years and > 2000 scans of CMR experience using a standardized approach for short-axis slice positioning [17].

Common imaging parameters for T1 mapping included slice thickness 8 mm, field-of-view 360 mm, echo time 1.07–1.22 ms, repetition time 2.14–2.44 ms, flip angle 20° for 3 T Philips Ingenia or 35° for the other systems).

For the assessment of global left ventricular (LV) parameters, standard breath-hold balanced stead-state free-precession (bSSFP) images were acquired in 2-chamber and 4-chamber long-axis views.

### Image acquisition – validation step

A second set of healthy subjects and patients with cardiac amyloidosis (ATTR, diagnosed by comprehensive workup including right-ventricular endomyocardial biopsy and/or DPD scintigraphy) underwent MOLLI 5 s(3 s)3 s either on a 1.5 T (Ingenia, Philips Healthcare; healthy subjects: 7 male, 7 female; 53 ± 7 years; cardiac amyloidosis patients: 16 male, 9 female; 66 ± 10 years) or a 3 T (Ingenia, Philips Healthcare; healthy subjects: 11 male, 5 female; 54 ± 3 years; cardiac amyloidosis patients: all 13 male; 68 ± 12 years).

Common CMR imaging parameters included slice thickness 10 mm, field-of-view 300 mm, echo time 1.17 ms, repetition time 2.34 ms, flip angle 35° at 1.5 T or 20° at 3 T.

For the assessment of global LV parameters, standard breath-hold bSSFP images were acquired in short-axis stacks.

### Z-score mapping

Image analysis was performed using a research version of cvi^42^ (Circle Cardiovascular Imaging Inc., Calgary, Canada) equipped with a prototype Z-score mapping module.

For Z-score mapping, average values of septal LV myocardial T1 were derived from the T1 maps generated by the CMR systems of all healthy control subjects and patients with cardiac amyloidosis by manual delineation of endocardial and epicardial contours with a standard segmentation tool. The software was set to automatically exclude the outer 20% of subendocardial and subepicardial layers in order to minimize partial volume effects from adjacent blood pool or extra-myocardial tissues as recommended [14]. Based on the results, mean and SD were calculated separately for each MOLLI variant and each group of subjects on each system.

In a second step, Z-score maps were generated for each T1 map from healthy subjects (evaluation step) and cardiac amyloidosis patients (validation step) based on the mean and SD values derived from corresponding T1 maps of the healthy subjects using the prototype Z-score module. Essentially, the Z-score module calculates the Z-score by

$$ \mathrm{Z}-\mathrm{score}=\left(\mathrm{T}1-\mathrm{mean}\right)/\mathrm{SD} $$

for each pixel of a T1 map, where T1 is the observed pixel value on the T1 map, and mean and SD are the mean and standard deviation of native myocardial T1 obtained from a group of healthy subjects with the given MOLLI variant and CMR system.

From the results, a Z-score map is generated where the intensity of each pixel corresponds to the Z-score of the T1 value of the corresponding pixel on the T1 map. As pixel intensities on DICOM images must have integer values, Z-score values are multiplied by 100 for visualization and storage (for example, a Z-score of 1.5 would be presented as 150). The Z-score map is shown and stored using a diverging colour scheme [18] (see Additional file 1) that was generated using Colorbrewer 2.0 (http://colorbrewer2.org; Cynthia Brewer, Mark Harrower and The Pennsylvania State University).

Finally, average Z-scores of septal LV myocardial T1 were derived from all Z-score maps by copying the endocardial and epicardial contours from the T1 maps using the same standard segmentation tool as described above. Average Z-scores were noted for each map, and mean and SD of Z-scores were calculated for each MOLLI variant for healthy subjects and for cardiac amyloidosis patients as described above for T1.

Global LV parameters (end-diastolic volume, ejection fraction, mass) were assessed from the cine images using biplane long-axis (evaluation step) or multi-slice short-axis (validation step) analysis.

### Statistical analysis

A normal distribution of the results was verified for each group of results using the Shapiro-Wilk Test and the Kolmogorov-Smirnov Test. In the evaluation step, ANOVA was used to test for the presence of significant differences. This was done separately for the native T1 results from 1.5 T and 3 T. If Levene’s test did not show homogeneity of variance, Welch’s ANOVA was performed. Afterwards a Bonferoni post-hoc analysis was performed to conduct multiple comparisons.

Independent t-tests were used to make comparisons between the two field strength using the same MOLLI from the same vendor.

In the validation step, independent t-tests were used to make comparisons in-between the two groups of healthy subjects and in-between the two cardiac amyloidosis patient groups as well as between healthy subjects and cardiac amyloidosis patients at corresponding field strengths. In case of significant differences, a power analysis was performed using the software program G*Power (Version 3.1) in order to estimate the statistical power of the results. If results were non-significant, an equivalence test (two one sided t-tests, TOST) was performed in the statistics software RStudio (Version 1.2.1335, 2009–2019 RStudio, Inc., Boston, Massachusetts, USA).

For the assessment of sensitivity and specificity, ranges of normal were defined as.

$$ \left(\mathrm{mean}-2\mathrm{SD}\right)\ \mathrm{to}\ \left(\mathrm{mean}+2\mathrm{SD}\right) $$

from the healthy subject data. Besides power analysis and equivalence tests, all statistical analysis was performed using SPSS (version 24, Statistical Package for the Social Sciences (SSPS), International Business Machines, Inc., Armonk, New York, USA).

## Results

### Evaluation step

**Fig. 1:**
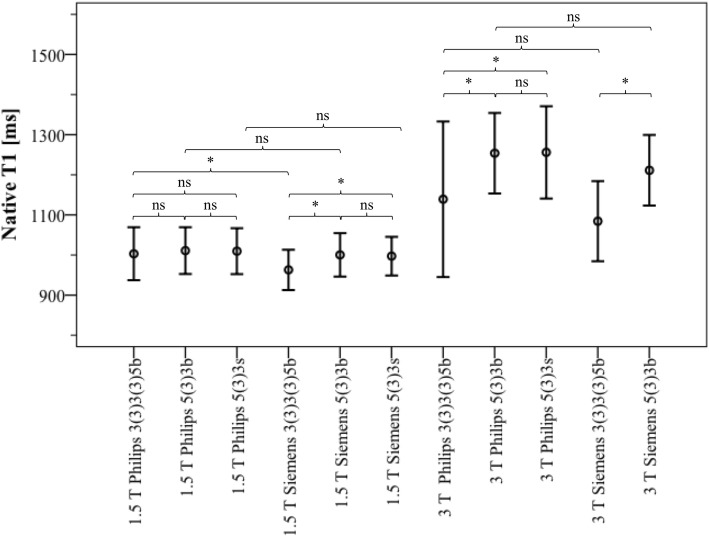
Native myocardial T1 (mean ± 2SD indicating 2.3rd/ 97.7th percentile) and results of Bonferroni post-hoc analysis in healthy subjects at different sites with different CMR systems, field strengths, and MOLLI schemes (for tabular data see Additional file 1). * = *p* < 0.05, ns = non-significant

**Fig. 2:**
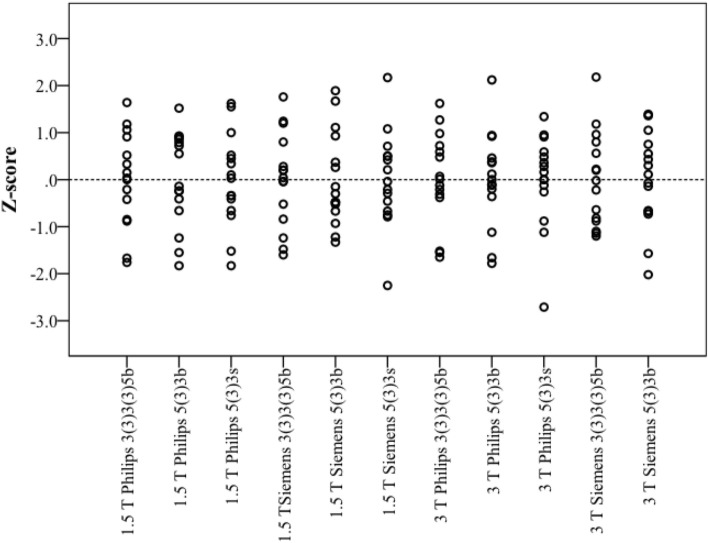
Z-score values of native T1 from healthy subjects (*n* = 15) at different sites with different CMR systems, field strengths, and MOLLI schemes

Table 1 provides global LV parameters of the healthy subjects as derived from cine CMR images. Figure 1 shows the results of cardiac T1 mapping in healthy subjects at different sites with different CMR systems, field strengths, and MOLLI schemes (for tabular data see Additional file 1) including results of Bonferroni post-hoc analysis. Both Shapiro-Wilk tests and Kolmogorov-Smirnov tests were performed and confirmed normal distribution of all T1 results in the healthy subjects. As expected, there were significant differences between mean native myocardial T1 values derived from different field strengths, manufacturers, and MOLLI schemes using both the classic ANOVA for 1.5 T and the Welch’s ANOVA for 3 T (*p* < 0.001, respectively). Independent t-tests showed significant differences between 1.5 T and 3 T for all comparisons made (always p < 0,001). While most SDs amounted to < 5% of mean T1 (≤33 ms at 1.5 T and ≤ 58 ms at 3 T), SD of MOLLI 3–3-5b data from the Philips system at 3 T was 97 ms (8.5% of mean T1), without any identifiable technical reason for the high variance of T1 values acquired with this specific combination. Figure 2 presents the corresponding Z-score values for the healthy subjects. As expected (proving the validity of the approach), mean Z-scores of healthy subjects were at or closely to 0.0 and ranged within − 2.71 to + 2.17.

**Table 1:** Essential characteristics and global left ventricular (LV) parameters derived from cardiovascular magnetic resonance (CMR) cine images in the evaluation step (healthy subjects) and validation step (healthy subjects and cardiac amyloidosis patients). Age and LV parameters are given as mean ± standard deviation. EDV = end-diastolic volume; EF = ejection fraction

	Subjects	Field strength	N	Age (years)	Male/ female	LV EDV (ml)	LV mass (g)	LV EF (%)
Evaluation	Healthy	1.5 T & 3 T	15	24 ± 4	7 / 8	187 ± 23.7	87 ± 20.9	62 ± 3.7
Validation	Healthy	1.5 T	14	53 ± 7	7 / 7	161 ± 18.3	81 ± 15.2	60 ± 3.6
	Healthy	3 T	16	54 ± 3	11 / 5	170 ± 29.8	91 ± 24.1	62 ± 2.7
	Amyloidosis	1.5 T	25	66 ± 10	16 / 9	179 ± 35.4	178 ± 53	50 ± 11.2
	Amyloidosis	3 T	13	68 ± 12	13 / 0	165 ± 32.6	167 ± 40	52 ± 13.7

**Fig. 3:**
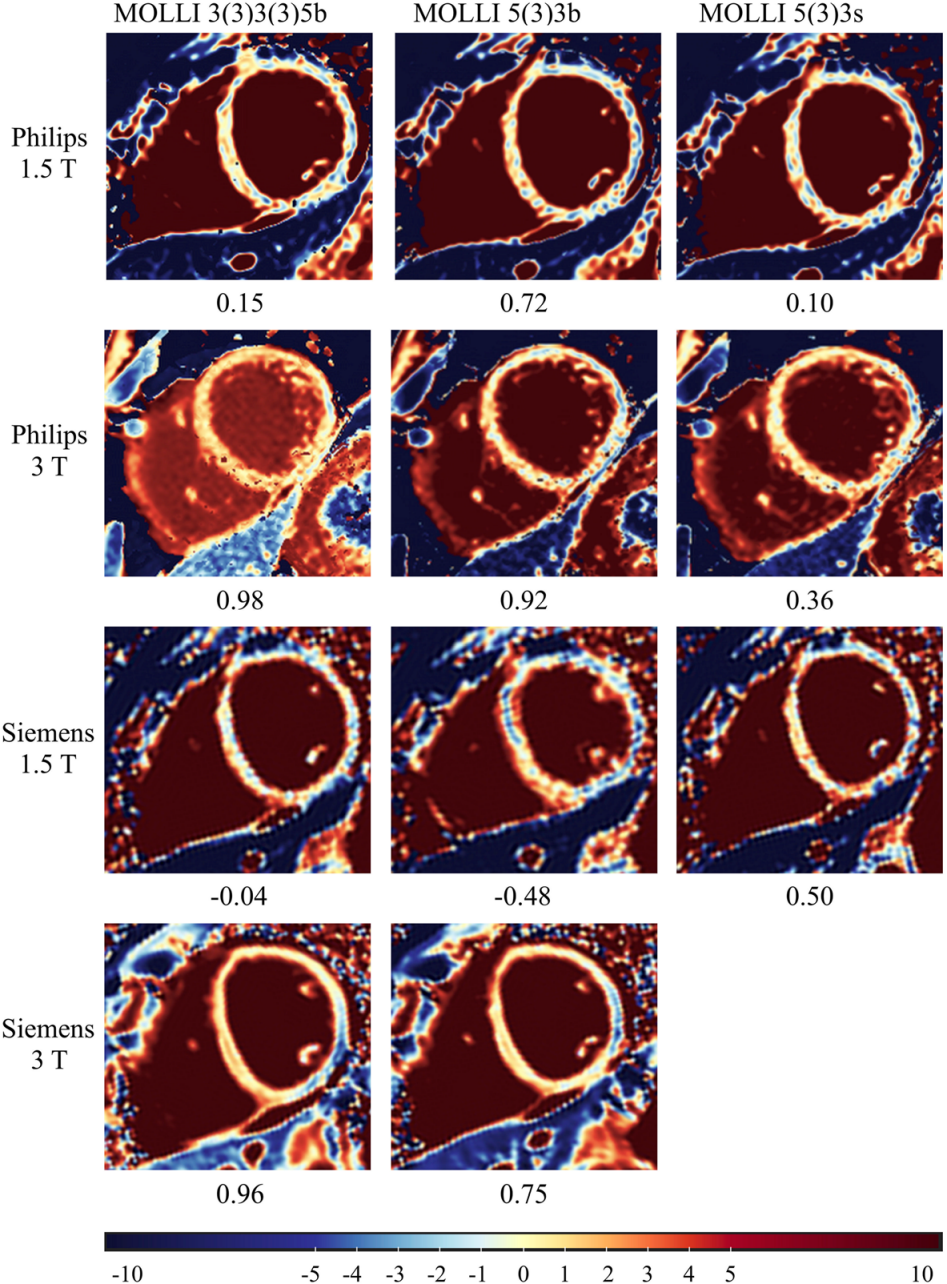
Full set of Z-score maps from a 22-year-old healthy female with corresponding Z-scores of native septal myocardium and diverging colour scale

In contrast to the T1 results, there was no significant difference detectable between the Z-scores derived from different field strengths, manufacturers, and MOLLI schemes using ANOVA (*p* = 1.0). A typical set of Z-score maps from one healthy subject is presented in Fig. 3.

### Validation step

**Fig. 4:**
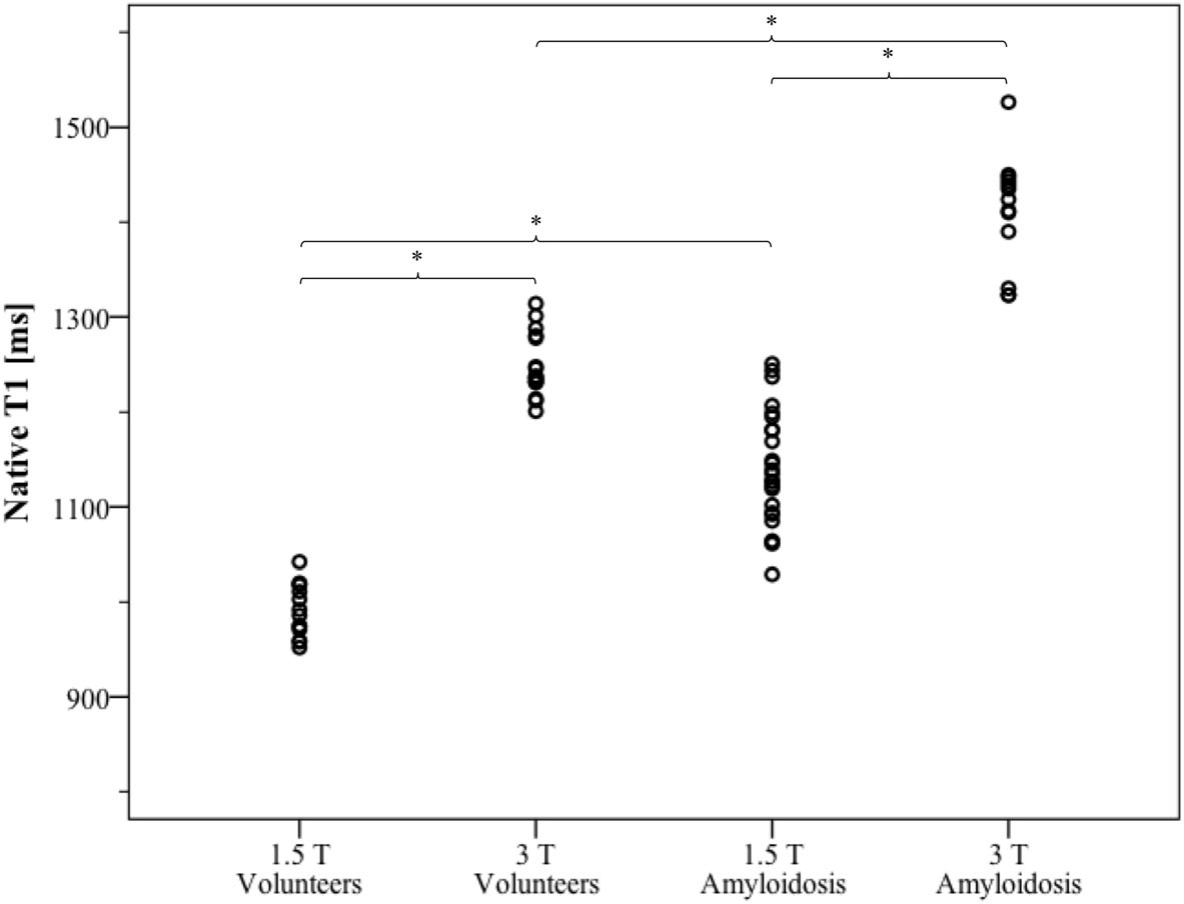
Native myocardial T1 of healthy subjects at 1.5 T (*n* = 14) or 3 T (*n* = 16) and cardiac amyloidosis patients at 1.5 T (*n* = 25) or 3 T (*n* = 13). Acquisition scheme: MOLLI 5(3)3b, * = *p* < 0.05

**Fig. 5:**
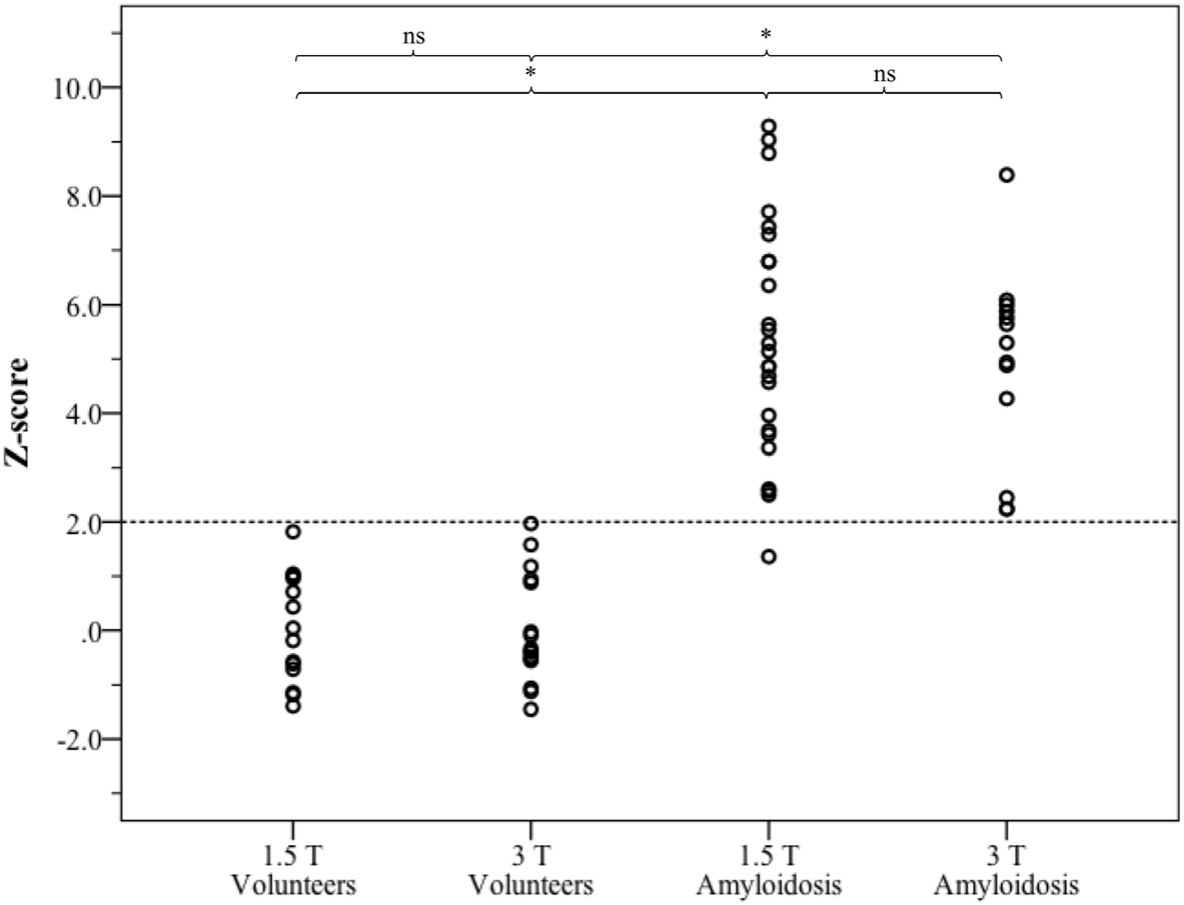
Z-score values of native T1 from healthy subjects at 1.5 T (*n* = 14) or 3 T (*n* = 16) and cardiac amyloidosis patients at 1.5 T (*n* = 25) or 3 T (*n* = 13). * = *p* < 0.05, ns = non-significant

**Fig. 6:**
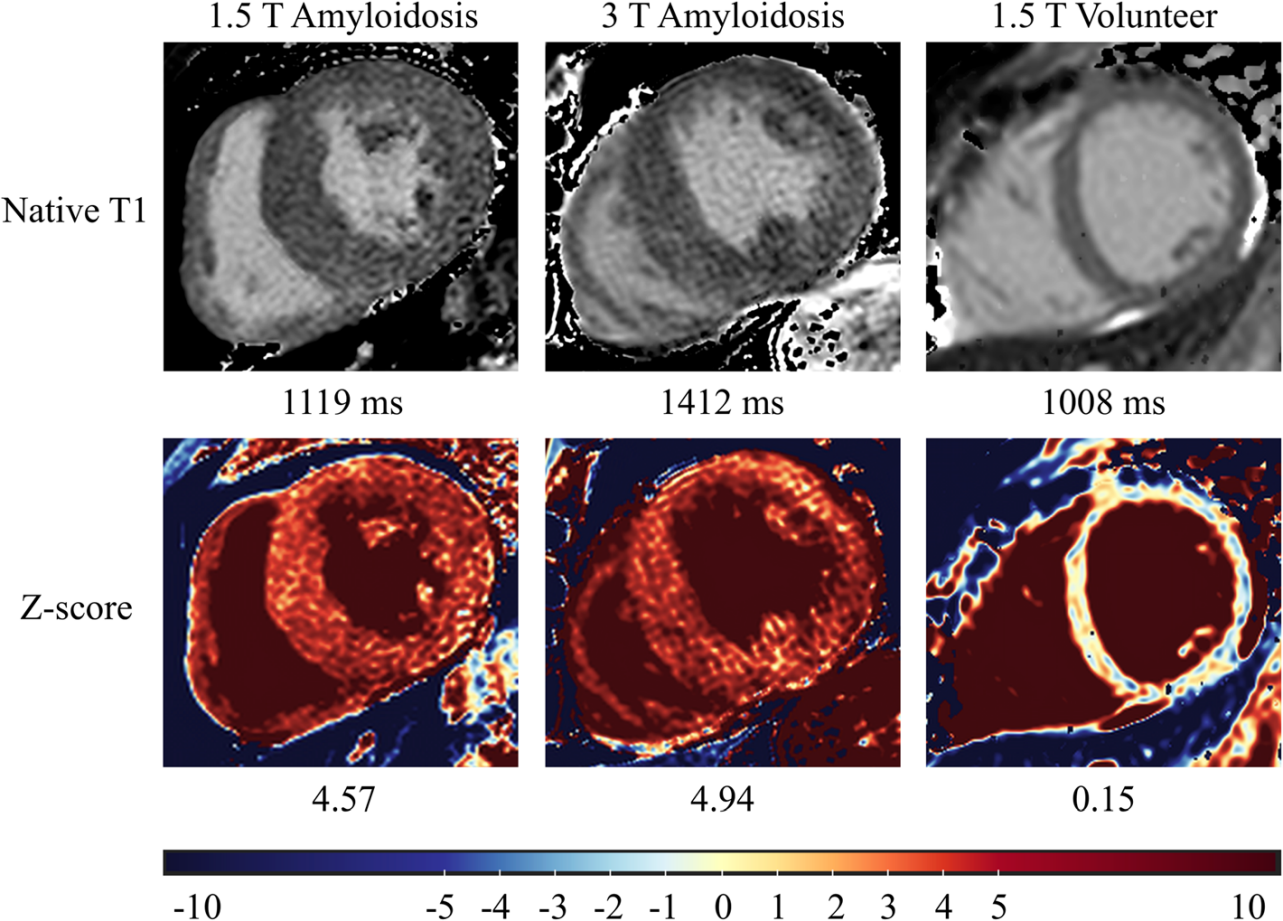
Examples of Z-score maps and corresponding T1 maps from two cardiac amyloidosis patients and one healthy subject. Left: 66-year-old male patient at 1.5 T,.mid: 78-year-old male patient at 3 T, right: 22-year-old healthy female at 1.5 T

As for the evaluation step, Table 1 provides global LV parameters as derived from cine CMR for both healthy subjects and cardiac amyloidosis patients. The results for T1 and Z-score analyses from healthy subjects and cardiac amyloidosis patients are presented in Figs. 4 and 5, respectively. As expected, patients with amyloidosis exhibited significantly higher myocardial T1 values than healthy subjects at the same field strength (*p* < 0.001, power 1.0). Native T1 was also different between 1.5 T and 3 T in both healthy subjects and cardiac amyloidosis patients (p < 0.001, power 1.0). Based on Z-score mapping using a threshold of Z = 2, amyloidosis was detected with the same sensitivity (96% at 1.5 T, 100% at 3 T, respectively) and specificity (100% at both 1.5 T and 3 T, respectively) as with T1 mapping, and the difference between healthy subjects and cardiac amyloidosis patients remained significant at both field strengths (p < 0.001 with a power of 1.0, respectively). In contrast no significant difference was observed for healthy subjects (*p* = 0.985) or patients with cardiac amyloidosis (*p* = 0.552) between results from 1.5 T or 3 T. For Z-scores from healthy subjects at different field strength, TOST verified equivalence at epsilon = 0.75 (*p* = 0.03, 95% TOST interval − 0.62 to 0.63). For cardiac amyloidosis patients from different field strength, TOST did not show equivalence at the same epsilon level of 0.75 (*p* = 0.309, 95% TOST interval − 0.69 to - 1.53) but at epsilon = 1.6 (epsilon = magnitude of region of similarity). Figure 6 shows examples of Z-score maps of cardiac amyloidosis patients at 1.5 T and 3 T.

## Discussion

Our results indicate that Z-score mapping might overcome the limitations of T1 mapping that are related to confounding effects of CMR hardware and software. The use of Z-score mapping should be further explored as a standardization tool for quantitative mapping of native relaxation times in the myocardium.

In cardiac applications, changes of myocardial tissue composition lead to changes of myocardial T1, which can be detected by established T1 mapping methodology with high reproducibility. Yet, due to the complexities of the hardware and software components involved, absolute numbers of normal and abnormal T1 differ between CMR systems and imaging centers, limiting the interchangeability of results. In this study we tested whether the transformation of native T1 values into Z-scores based on prior knowledge of normal ranges generated with a given combination of hardware/ software could eliminate site-specific differences of results.

In conventional T1 mapping, confounding effects of field strength, system design, and pulse sequence scheme are minimized post-hoc by interpreting the results in the light of local reference values. In Z-score mapping, this step is integrated into the image processing in order to make the results directly comparable between cohorts of different reference ranges. In the evaluation part of this study, Z-score mapping was applied as an additional post-processing step to a variety of MOLLI T1 data sets (native T1 maps) that were acquired with different hardware/ software combinations in a group of healthy subjects. While conventional analysis of the T1 maps showed the expected differences in myocardial T1 based on field strength and MOLLI scheme [12], analysis of Z-score maps yielded homogenous results without significant differences between the different sources. In the validation part of the study, Z-score mapping was applied to MOLLI T1 data sets from healthy subjects and cardiac amyloidosis patients acquired at 1.5 T or at 3 T in order to assess the diagnostic accuracy of Z-score mapping for differentiating normal from abnormal T1 behavior. Analysis of Z-score maps differentiated cardiac amyloidosis from normal myocardium with the same sensitivity and specificity as conventional T1 analysis. However, while the spectrum of T1 results depended largely on field strength, results of Z-score maps showed no significant differences between patients studied at 1.5 T vs. 3 T, or between healthy subjects studied at 1.5 T vs. 3 T. Thus, Z-score mapping allowed for directly comparing results of T1 measurements across different hardware/ software combinations including different field strengths.

The variation of T1 results is reflected by the SD of the mean for a group of measurements, and has been used as a marker for the reproducibility of T1 measurements within groups of healthy subjects [19]. Based on this parameter, there were some differences in diagnostic performance between different hardware/ software combinations in the evaluation part of this study. While SDs ranged from 24 to 33 ms at 1.5 T and from 44 to 58 ms in four combinations at 3 T, the SD of one particular combination at 3 T amounted to 97 ms. While it could be expected that the 3–3-5 MOLLI scheme performed worse than 5–3 schemes at 3 T due to insufficient recovery times (in relation to myocardial T1 at 3 T) between subsequent inversion experiments, it remains unclear why this was the case on one 3 T CMR system but not on the other that were used. We could not identify any external confounders such as differences in heart rate of the healthy subjects during the different acquisitions. This phenomenon allowed us to study the impact of variations in the performance of the underlying acquisition strategies on Z-score results. If a site records a large SD of native T1 when generating normal data for its T1 measurements, this translates into a wide normal range. When this normal range is then applied in clinical routine, very high or very low T1 results will be required to qualify as “abnormal” at this site. In other words, T1 measurements will have a lower sensitivity for detecting disease at this site as compared to T1 measurements from sites with lower SD within control measurements. In Z-score mapping, the high SD of the specific 3–3-5 variant at 3 T resulted in a shift of the Z-scores and the corresponding color zones, visualizing the reduced discriminatory power of this variant. Thus, differences in sensitivity of a T1 mapping acquisition scheme are passed-on to Z-score maps, or in other words: Z-score mapping does not enhance the diagnostic performance of a T1 mapping acquisition strategy. At the same time, the obvious effects of the normal range on the diagnostic value of both standard T1 mapping and Z-score mapping underline the importance of operating with optimized acquisition schemes and with normal values that are carefully generated and valid for a specific site.

From a clinical perspective, Z-score results have to be interpreted in the light of the given clinical question. As they represent biological continuous data, there is no per-se cut-off value between “normal” and “disease”, and no predefined maximum value. Instead, ranges of Z-scores have to be established for different disease entities, and there will be overlap between Z-scores between diseases with mild effects on T1 and normal, corresponding to the underlying T1 behavior. Z-score mapping has the potential to enhance the ability of T1 mapping to detect subtle changes in these “low-magnitude pathologies” [14] by allowing for generating patient-specific Z-score maps based on granular age- and gender-specific databases of normal T1 in an automated fashion. Future studies will be necessary to generate such databases (e.g. from population-based studies of healthy subjects) and implement automated Z-score mapping. However, rigorous standardization of analysis procedures is necessary in order to avoid magnification of small differences by applying Z-scores from low-variation normal data (e.g. large septal mid-cavity regions of interest (ROIs)) to situations of higher variation (e.g. small ROIs) or from other mean levels (e.g. apical orientation).

As does T1 mapping, Z-score mapping allows for both ROI-based numerical analysis and colour-based visual assessment. In order to facilitate visual detection of abnormal myocardial tissue, a diverging colour scale was implemented in the Z-score mapping module rather than a rainbow color scale [18]. The current recommendations for clinical applications of T1 mapping demand that “look up tables are set according to site-specific ranges of normal” in order to be applied for T1 maps [14]. Since the use of specific ranges of normal for a given hardware/ software combination is at the center of Z-score mapping, the Z-score approach inherently fulfills this requirement, and the proposed color scheme might be usable without further adjustments when applied to other data sets with their respective mean and SD values.

In our study, only variants of MOLLI were available for comparison. In principle, Z-score mapping should equally be applicable to T1 data from other acquisition methods including shortened MOLLI (ShMOLLI) [9], saturation recovery single-shot acquisition (SASHA) [10], saturation pulse prepared heart rate independent inversion recovery (SAPPHIRE) [11], and others, provided that the normal behavior (mean, SD) of that method is known. Based on the results of the evaluation part of this study, similar effects would be expected when applying Z-score mapping to data from any of those acquisition methods as for going from one MOLLI scheme to another, i.e. homogenization of the levels of the results while maintaining sensitivity and specificity of the respective technique [12, 20]. Furthermore, the general considerations discussed above on behavior, comparability, and analysis of T1 mapping data apply equally to data from T2 mapping. Thus, Z-score mapping might also be useful for standardizing the analysis of T2 maps from different sources [21, 22]. However, this was not investigated in this project and requires further studies.

Another potential way of standardizing results from T1 mapping involves the use of standardized phantoms. In this approach, phantoms with predefined, stable T1 values [23] might be scanned with a site-specific T1 mapping variant. The results could then be standardized using linear or non-linear correction algorithms to reach either the “true” T1 of the phantom as provided by the manufacturer, or an agreed-upon “standard” T1 (e.g. 1000 ms for phantoms whose T1 values correspond to those of normal myocardium). In contrast to the phantom approach, Z-score mapping does not require additional (and costly) hardware, and standardizes in relation to the actual biological tissue of interest rather than an external body. On the other hand, phantom measurements are able to detect systematic changes of magnetic system behavior over time (“drift”), which might be missed by Z-score mapping (and conventional T1 mapping) unless normal ranges are verified or reassessed on a regular basis. Thus, regular phantom measurements remain an important tool for quality control [24] even if Z-score mapping is used instead of standard T1 analysis.

For this study, a condition with large-magnitude biological changes (cardiac amyloidosis) was chosen to test the performance of Z-score mapping as compared to standard T1 analysis. While the results of our study demonstrated no loss of diagnostic accuracy with the use of Z-score analysis, further studies are necessary to assess the performance of this approach in small-magnitude biological changes (e.g. diffuse myocardial fibrosis). In order to enhance diagnostic accuracy in these scenarios, large multi-dimensional normal databases might be used to generate maps of age- and sex-matched Z-scores for individual patients. As another limitation of our study, Z-score mapping was not tested in low-T1 myocardial diseases (i.e. Fabry’s, iron overload). Even if its diagnostic behavior should not differ in these situations from high-T1 diseases, future studies in cohorts of such patients are warranted to verify the validity of the Z-score approach in these scenarios.

## Conclusions

In summary, the use of Z-score mapping for quantifying native myocardial T1 provided consistent results without significant differences between data from different field strengths, CMR systems, or MOLLI variants in healthy subjects. Z-score mapping identified patients with cardiac amyloidosis with the same diagnostic accuracy as conventional T1 analysis. Z-score mapping holds the potential to allow for standardized quantification and reporting of native myocardial T1 across different CMR hardware/ software combinations, and for comparing MOLLI T1 results from different CMR systems and centers in both research and clinical routine.

## Supplementary information

**Additional file 1.** Color scheme and tabular results.

## Data Availability

For additional information see supplemental material. The image datasets used and analysed during the current study are available from the corresponding author on reasonable request.

## Abbreviations

ATTR: Amyloid transthyrein
bSSFP: Balanced steady state free precession
CMR: Cardiovascular magnetic resonance
ECG: Electrocardiogram
EDV: End-diasatolic volume
EF: Ejection fraction
LGE: Late gadolinium enhancement
LV: Left ventricle/left ventricular
MOLLI: Modified Look-Locker inversion recovery
ROI: Region of interest
SAPPHIRE: Saturation pulse prepared heart rate independent inversion recovery
SASHA: Saturation recovery single-shot acquisition
ShMOLLI: Shortened modified Look-Locker inversion recovery
STIR: Short tau inversion recovery
