# Supplementary material for: Z-score mapping for standardized analysis and reporting of cardiovascular magnetic resonance modified Look-Locker inversion recovery (MOLLI) T1 data: Normal behavior and validation in patients with amyloidosis
Source: J Cardiovasc Magn Reson. 2020 Jan 20;22:6. doi: 10.1186/s12968-019-0595-7 (PMC6970284; doi:10.1186/s12968-019-0595-7)
Supplement: Supplementary file 1 — Additional file 1. Color scheme and tabular results. [file 12968_2019_595_MOESM1_ESM.docx]

**Supplemental material**

**Color scheme**

Created with Colorbrewer 2.0 (Cynthia Brewer, Mark Harrower and The Pennsylvania State University)

Link: [http://colorbrewer2.org/ - type=diverging&scheme=RdYlBu&n=11](http://colorbrewer2.org/#type=diverging&scheme=RdYlBu&n=11)

RGB values:

5 (165,0,38)

4 (215,48,39)

3 (244,109,67)

2 (253,174,97)

1 (254,224,144)

0 (255,255,191)

-1 (224,243,248)

-2 (171,217,233)

-3 (116,173,209)

-4 (69,117,180)

-5 (49,54,149)

**Tables**

Table 1

|  | **MOLLI scheme** | | | | | |
| --- | --- | --- | --- | --- | --- | --- |
|  | **3(3)3(3)5b** | | **5(3)3b** | | **5(3)3s** | |
|  | **mean±SD** | **normal  range** | **mean±SD** | **normal  range** | **mean±SD** | **normal  range** |
| **1.5 T Philips** | 1003±33 | 937 – 1069 | 1011±29 | 953 – 1069 | 1010±29 | 952 – 1068 |
| **1.5 T Siemens** | 963±25 | 913 – 1013 | 1000±27 | 946 – 1054 | 997±24 | 949 – 1045 |
| **3 T Philips** | 1139±97 | 945 – 1333 | 1254±50 | 1154 – 1354 | 1256±58 | 1140 – 1372 |
| **3 T Siemens** | 1084±50 | 984 – 1184 | 1211±44 | 1123 – 1299 | - | - |

Part 1 of study (evaluation): Native myocardial T1 [ms] from healthy volunteers (n=15). Normal ranges were defined as ±2 standard deviations (SD) from the mean.

Table 2

|  | **MOLLI scheme** | | | | | |
| --- | --- | --- | --- | --- | --- | --- |
|  | **3(3)3(3)5b** | | **5(3)3b** | | **5(3)3s** | |
|  | **mean±SD** | **min – max** | **mean±SD** | **min – max** | **mean±SD** | **min – max** |
| **1.5 T Phillips** | 0.00±1.00 | -1.76 – 1.64 | 0.00±1.01 | -1.83 – 1.52 | -0.02±0.99 | -1.83 – 1.62 |
| **1.5 T Siemens** | 0.00±1.01 | -1.60 – 1.76 | 0.01±1.00 | -1.33 – 1.89 | 0.01±1.00 | -2.25 – 2.17 |
| **3 T Phillips** | 0.00±1.00 | -1.65 – 1.62 | 0.00±1.00 | -1.78 – 2.12 | 0.00±0.99 | -2.71 – 1.34 |
| **3 T Siemens** | 0.01±1.00 | -1.20 – 2.18 | 0.00±1.00 | -2.02 – 1.39 | ~~-~~ | ~~-~~ |

Part 1 of study (evaluation): Z-scores of native myocardial T1 from healthy volunteers (n=15). SD = standard deviation; min = minimum value obtained; max = maximum value obtained.

Table 3

|  | **group** | **mean±SD** | **normal range** | **min – max** |
| --- | --- | --- | --- | --- |
| **1.5 T Philips** | volunteers | 991±28 | 935 – 1047 |  |
|  | patients | 1141±61 |  | 1029 – 1251 |
| **3 T Phillips** | volunteers | 1249±33 | 1183 – 1315 |  |
|  | patients | 1412±59 |  | 1323 – 1526 |

Part 2 of study (validation): Native myocardial T1 [ms] from healthy volunteers at 1.5 T (n=14) or 3 T (n=16), and from patients with cardiac amyloidosis at 1.5 T (n=25) or 3 T (n=13). Normal ranges were defined as ±2 standard deviations (SD) from the mean. Min = minimum value obtained; max = maximum value obtained.

Table 4

|  | **group** | **mean±SD** | **min – max** |
| --- | --- | --- | --- |
| **1.5 T Philips** | **volunteers** | 0.02±0.99 | -1.39 – 1.82 |
|  | **patients** | 5.35±2.16 | 1.36 – 9.29 |
| **3 T Philips** | **volunteers** | 0.01±1.00 | -1.45 - 1.97 |
|  | **patients** | 4.93±1.77 | 2.24 – 8.39 |

Part 2 of study (validation): Z-scores of native myocardial T1 from healthy volunteers at 1.5 T (n=14) or 3 T (n=16), and from patients with cardiac amyloidosis at 1.5 T (n=25) or 3 T (n=13). SD = standard deviation; min = minimal value obtained; max = maximum value obtained.
